# Development of a Two Port Laparoscopic Appendectomy Technique at a Rural Hospital

**DOI:** 10.1155/2019/9761968

**Published:** 2019-05-19

**Authors:** Hugo J. R. Bonatti

**Affiliations:** ^1^University of Maryland Community Medical Group, Surgical Services, Easton, MD, USA; ^2^Meritus Surgical Specialists, Hagerstown, MD, USA

## Abstract

**Background:**

Laparoscopic appendectomy (LA) is most commonly performed using two 5-mm and one 10/12-mm ports. Various attempts to reduce the number and size of ports have been made and new technologies such as single port LA have been introduced. Appendix and mesoappendix are usually divided with a stapler or energy device with electrocautery, clips, and endoloop being cheaper options.

**Patients and Methods:**

This study includes 51 consecutive LAs performed at a rural hospital. Patients were divided into 4 groups: group 1 was the standard technique group (n=12), group 2 served as a “try-out” (n=12), group 3 served as feasibility group (n=12), and group 4 was the final patient cohort in which the optimized technique was preferably used (n=15).

**Results:**

Median age of the study cohort was 35.4 (range: 6.2-80.6) years, and 55% of patients were male. Whereas in G1 all patients had standard port placement (10/12-mm, 2x5-mm), in an increasing number of patients in G2-4 only two 5-mm ports and the 2.3-mm Teleflex minigrasper were inserted. Usage of staplers and/or energy devices was reduced from 100% in G1 to 20% in G4, and in the majority of cases both the appendix and the vascular pedicle were secured with an endoloop. The new technique did not add time to the procedure or total OR time. No stump-leaks or surgical site infections were encountered in this series, and there were no conversions to open surgery. Cost savings when not using a stapler or energy device are approximately 400$ per case; the minigrasper added approximately 200$ to the case.

**Discussion:**

LA with use of two ports and a portless needle grasper is feasible in the majority of cases and was associated with high patient satisfaction and excellent cosmetic results. Avoiding energy devices and staplers is cost saving; the endoloop securely controls appendix and mesoappendix.

## 1. Introduction

Multiple studies have shown that subsets of patients with acute appendicitis may be treated with antibiotics [1]. However, most surgeons consider that appendectomy is still a better option, and the vast majority of appendectomies today are done using a laparoscopic approach [2]. Laparoscopic appendectomy (LA) has multiple advantages over the open approach with less pain, faster recovery, and less scarring being the most important benefits. Most surgeons insert two 5-mm and one 10/12-mm ports, with the latter placed into the umbilicus. After the appendix and the mesoappendix are separated, the appendix and the vascular pedicle can be secured by various methods including staplers, clips, and energy devices such as harmonic scalpel, EnSeal, or LigaSure amongst many other options [3, 4]. Using an endoloop to tie off the appendix stump has been shown to be safe, and thus the use of a 10-mm port for the stapler can be avoided [5]. Various techniques to reduce the number [6, 7] and size of ports during LA have been published, and in children mini-instruments are commonly used [8, 9]. In adults use of a suture to suspend the appendix has been suggested by Roberts et al. [7]. Other authors have suggested single port appendectomy [10–12] and transvaginal appendectomy [13, 14], and even robotic assisted appendectomy has been done [15]. In the rural setting with limited resources and staff available, reduction of individuals involved in the procedure is desired. In addition, use of smallest size trocars to help avoid development of trocar site hernias is crucial as many patients need to return early to work, which in many cases is hard physical work such as that on farms. At the same time, reduction of costs per case has become a key issue in financial planning for rural hospitals as well as hospitals in developing countries [16, 17].

The aim of this study was to retrospectively analyze a series of LAs performed by a single laparoscopically trained surgeon in a rural hospital with the goal of stepwise optimizing the technique of LA towards a less invasive, less expensive, and equally safe procedure as the standard technique.

## 2. Patients and Methods

This is a retrospective analysis of all LAs performed by a single surgeon with fellowship training in minimal invasive surgery during a 30 month period. A total of 51 consecutive LAs were analyzed.

The endpoints to optimize the procedure were reduction in the number and size of ports, which should be accomplished in parallel to avoiding use of expensive instruments such as staplers and energy devices. At the same time it was crucial to provide highest patient safety and not to increase the total operative time or hospitalization rate.

The study was approved by the ethics committee. Patients' consent was obtained with regard to the alternative technique, i.e., the two port technique, that would be used if technically feasible. Data were obtained from electronic medical records (Meditech, EPIC) and a database was created using MS Excel. Data are displayed as percent of the population for discrete parameters and median with range for continuous parameters. Statistical analysis was done using MS Excel and SPSS. A p-value of <0.05 was considered statistically significant.

Four study groups were created according to the time of surgery within the study. Group 1 was the standard technique group (n=12), group 2 served as a “try-out” (n=12), group 3 served as feasibility group (n=12), and group 4 was the final patient cohort in which the optimized technique was preferably used (n=15).  Table 1 shows demographic and clinical data of the study population according to the four study groups.

Diagnosis of appendicitis was made by a history of typical right lower quadrant pain with or without concomitant nausea together with clinical examination with a positive McBurney's sign. Diagnosis was supported by ultrasound and/or CT-scan in all cases. Patients were started on antibiotics (ertapenem 1g or ampicillin/sulbactam 3g or ciprofloxacin 200mg in combination with metronidazole 500mg in case of penicillin allergy) as soon as diagnosis of acute appendicitis was made. The majority of patients were operated on between 4 and 16 hours after diagnosis was established depending on admission time and OR availability. Patients received an additional dose of antibiotics at time of induction of surgery, if waiting time for surgery exceeded recommended antibiotic redosing.

Per protocol, all patients with RLQ abscess formation and/or phlegmon involving the surrounding tissue were hospitalized and treated primarily with antibiotics. Those with abscess formation were evaluated together with interventional radiology, and a percutaneous drain was placed whenever possible. Patients underwent reimaging within 1-2 weeks, and those older than 50 years who did not have a recent colonoscopy were scheduled for a colonoscopy to rule out any additional pathology in the right hemicolon and/or terminal ileum. Interval LA was only done if patients continued to have RLQ pain or had fever or if imaging was suggestive for ongoing or recurrent appendicitis.

### 2.1. Surgical Technique

#### 2.1.1. Standard Technique

After induction of anesthesia, patients were prepped and draped. The abdomen was accessed with a Verres needle in the left upper quadrant (LUQ) at Palmer's point, and once pneumoperitoneum was established a 5-mm trocar was placed under visual control into the left lower quadrant (LLQ). The Verres needle was exchanged for another 5-mm trocar, and a 10/12-mm umbilical trocar was inserted. The 30-degree 5-mm camera was placed in the LUQ port. A window was created at the base of the appendix between the appendix and the vascular pedicle, and the two structures were divided using a stapler. The vascular pedicle was secured with an energy device such as a harmonic scalpel or EnSeal in few cases. The appendix was amputated and dropped into a 10-mm retrieval bag and removed through the umbilical port site.

#### 2.1.2. Optimized Technique Using Two 5-mm Ports and a Needle Grasper

**Figure 1:**
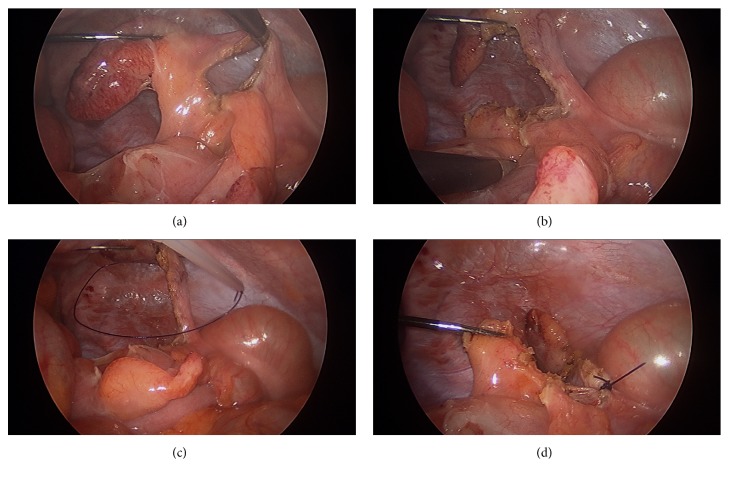
Laparoscopic appendectomy using two 5-mm ports and a needle grasper. (a) Phlegmonous appendix; window between appendix and mesoappendix. (b) The mesoappendix has been completely dissected off the appendix. (c) An endoloop is lassoed around the skeletonized appendix. (d) The appendix is tied at the base and amputated; the mesoappendix is lifted up to be secured with a second endoloop.

The abdomen was accessed with a 5-mm 1st entry port (Kii Fios First Entry Access System, Applied Medical, Rancho Santa Margarita, CA) in the LUQ, and pneumoperitoneum was established. A 5-mm port was placed into the umbilicus and a 2.3-mm minigrasper (MiniLap® Percutaneous Surgical System, Teleflex, Morrisville, NC) without trocar was inserted through a suprapubic 2-mm incision. The appendix was grasped with the minigrasper and pulled up. An L-hook or endoshears with electrocautery were used to create a window between appendix and mesoappendix (Figure 1(a)). The window was widened and the mesoappendix completely dissected off the appendix (Figure 1(b)). Endoloops were lassoed around the appendix and the mesoappendix, respectively, and tied (Figures 1(c) and 1(d)). The appendix was amputated at the base, dropped into a 5-mm retrieval bag, and removed through the LUQ or umbilical port site.

## 3. Results

The study includes 51 consecutive patients undergoing LA. Median age of the patients was 35.4 (range: 6.2 to 80.6) years; 55% were male. Clinical data according to the four groups are depicted in Table 1. No statistical significant differences were found for base characteristics such as gender, age, and outcomes including complications, operative time, and length of stay. Ten patients (19.6%) presented with perforated appendicitis; two patients had an interval appendectomy after they had a percutaneous drain for a pericecal abscess and ongoing RLQ pain. In one patient with breast cancer the appendix was removed for a mucocele [18]. One patient had chronic appendicitis with the appendix stuck to an aortobifemoral graft causing small bowel obstruction. No cases were done between midnight and 6 am with 44% of cases being started in the morning, 42% in the afternoon, and 14% between 6 pm and midnight. Nine patients had additional procedures (n=11) done including umbilical hernia repair (n=6), lysis of adhesions (n=4), and laparoscopic cholecystectomy (n=1). In two patients of group 1, including the patient with laparoscopic cholecystectomy, a fourth port was used, and in one case it was used to retract the liver as the appendix was caught in subhepatic adhesions.

**Figure 2:**
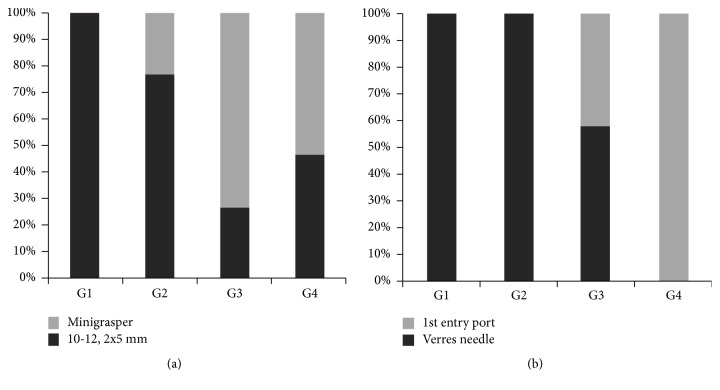
Operative data A. (a) Increasing usage of the minigrasper in groups 3 and 4. (b) Switch from Verres needle approach to Fios 1st entry port.

**Figure 3:**
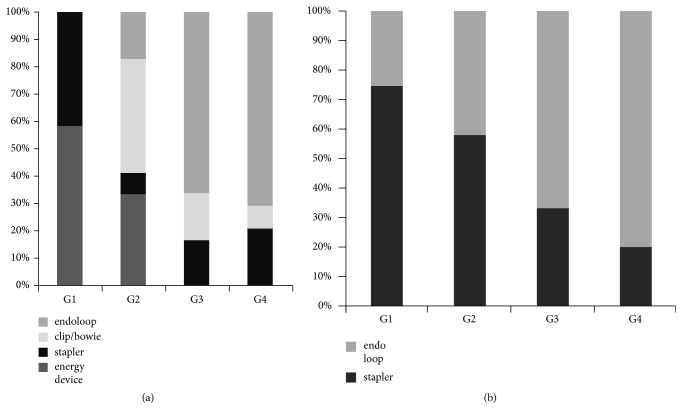
Operative data B: techniques to secure vascular pedicle and appendix. (a) The mesoappendix was initially secured with a stapler or energy device but in groups 3 and 4 mainly with an endoloop. (b) Appendix was initially mainly stapled off but in groups 3 and 4 mainly secured with an endoloop.

In G1, all patients had standard port placement (2x5-mm plus 10/12-mm); in an increasing number of patients in G2-4, only two 5-mm ports and the minigrasper without trocar were used (Figure 2(a)). In G3 and G4 the first entry port was increasingly used (Figure 2(b)). Usage of staplers and/or energy devices to secure appendix and mesoappendix was reduced from 100% in G1 to 20% in G4, and in the majority of cases appendix and vascular pedicle were secured with an endoloop (Figures 3(a) and 3(b)).

**Figure 4:**
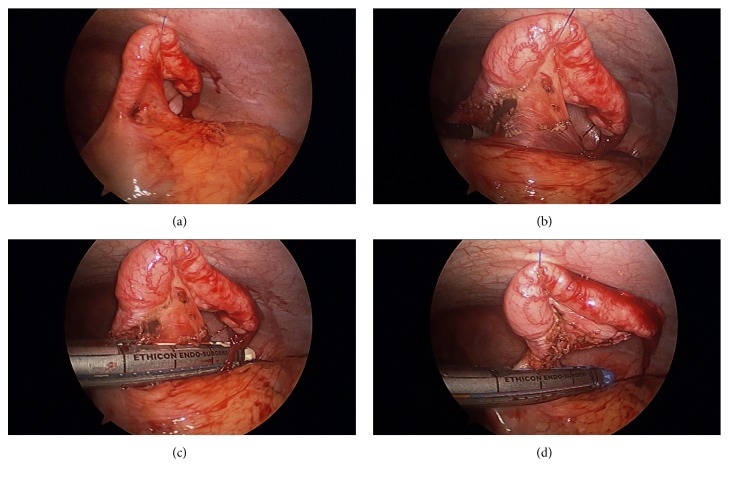
(a) The appendix is suspended to the abdominal wall using a Keith needle suture. (b) A window is created between appendix and mesoappendix. (c) The vascular pedicle is stapled. (d) The appendix is stapled at the base.

In one patient in group 2, LA was done with only two 5-mm ports and no minigrasper due to favorable anatomy with the appendix being adhered to the right lateral abdominal wall [6]. In another two port LA case, a 5-mm and 10/12-mm trocar were inserted and the appendix was suspended with a Keith needle in the RLQ (Figures 4(a), 4(b), 4(c), and 4(d)).

No stump-leaks or surgical site infections were encountered in this series and there were no conversions to open surgery. Half of cases were done as same-day surgeries, 32% of patients were placed into extended recovery, and 18% of patients required admission.

Cost savings when avoiding energy devices and/or staplers were approximately 400$ per case. The portless minigrasper adds approximately 200$ to the case.

## 4. Discussion

LA can be done in the majority of cases with two 5-mm ports and a minigrasper. Two port LA has been shown to be feasible using various techniques and in most series one 5-mm and a 10-mm port were used with an additional instrument such as suspension ties, suture passer, or minigraspers inserted to lift the appendix up [7, 8, 19, 20]. In many cases, the authors still use expensive tools such as energy devices and/or staplers [19]. The use of ties such as an endoloop has been shown to be safe. Endoloops cost only a fraction of the above listed instruments [5].

Although nonoperative management of acute appendicitis has been shown to be a viable option for some patients [1], appendectomy is still considered the standard treatment by most surgeons and laparoscopy is the preferred approach [2, 17, 21]. The diagnostic pathway of RLQ pain has dramatically changed during the past decade in many countries including the USA. Surgeons are consulted in the majority of cases after diagnosis of acute appendicitis by CT-scan is done in the emergency room. The decision to operate or not to operate on these patients should remain in the hands of the consulted surgeon; however, this decision may be made in the future by ER physicians, and surgery is challenged by the alternative of avoiding a procedure at all. LA certainly offers a better choice for patients when compared to open appendectomy, but there are several options to improve the standard operative technique aiming for a less invasive procedure including mini-incision and hybrid appendectomy [22, 23]. SILS, NOTES, and robotic assisted appendectomy have been shown to be feasible but are not universally available, increase costs, and do not make incisions smaller [11, 14, 24]. Single and two port techniques including the transumbilical appendectomy seem to have advantages over these techniques and are cost effective [7, 10]. Also the two port appendectomy with use of a suture passer to handle the appendix is an appealing and inexpensive alternative [19].

In our series we not only aimed to reduce number of ports but also tried to avoid insertion of a 10/12-mm trocar. Trocar site hernias most commonly develop at the umbilicus and are almost always associated with trocars with a diameter >10 mm [25]. In order to be able to remove the appendix from a smaller incision, we routinely completely skeletonize the appendix and we preferably remove the specimen through the LUQ port site. Of note, most 5-mm retrieval bags are accommodated by the Fios 1st entry but not by many other 5-mm trocars. Access to the abdominal cavity using this insufflating port at Palmer's point is safe and faster than Verres needle insertion followed by optical trocar placement or open access using the Hassan technique. In order to keep number and size of incision to a minimum, we propose that, rather than having fixed ports ready for access, the procedure should be started with a 5-mm port, and the abdominal cavity should be explored and then surgeons are able to decide the next steps including additional port placements based on anatomical findings and the appearance of the appendix. With this, protocol insertion of a 10/12-mm trocar can be avoided in most cases; however, no staplers can be used to secure appendix or mesoappendix. Endoloops, 5-mm clips, electrocautery, and, if hemostasis is difficult, an energy device such as the harmonic scalpel, EnSeal, or LigaSure device may be used as they can be inserted through a 5-mm port.

The 2.4-mm Teleflex minigrasper (Teleflex Morrisville, NC, USA) is inserted without a trocar and has been shown in multiple series to be able to replace a conventional trocar based grasper [26]. In this series, in the majority of cases, a minigrasper with a pistol grip was used, which is significantly more versatile than the older thumb-grip version. A cheap and simple technique for LA using a suture passer has been recently reported by Donmez [19].

We believe that patients benefit from an approach attempting to cause minimal surgical trauma by use of less and smaller incisions. This is a small series from a single institution, and results need to be reproduced by others. Nevertheless, the goal to achieve less pain, faster recovery, and a better cosmetic result is appealing and should encourage surgeons to try this approach. This is particularly true for a relatively simple and frequent procedure such as LA.

## Figures and Tables

**Table 1:** Demographic and clinical data.

	Group 1	Group 2	Group 3	Group 4	
n patients	12	12	12	15	ns
median age (range) (years)	51.1 (13.4-71.4)	44.8 (14-76.8)	36.9 (6.2-68)	31.9 (15.7-80.6)	ns
male (%)	58%	58%	75%	50%	ns
median OR time (min)	83 (68-110)	81 (74-140)	84 (58-152)	72 (62-137)	ns
additional procedures (%)	25%	17%	25%	7%	ns
perforated appendicitis (%)	17%	25%	8%	27%	ns
chronic/other appendicitis (%)	8%	8%	25%	20%	ns
Minigrasper (%)	0%	17%	66%	53%	p<0.05
stapler/energy device (%)	100%	58%	33%	20%	p<0.05

## Data Availability

The data used to support the findings of this study are available from the corresponding author upon request. However, patients' data cannot be made available due to HIPA regulations.
